# Thrombin-induced cytoskeleton dynamics in spread human platelets observed with fast scanning ion conductance microscopy

**DOI:** 10.1038/s41598-017-04999-6

**Published:** 2017-07-06

**Authors:** Jan Seifert, Johannes Rheinlaender, Florian Lang, Meinrad Gawaz, Tilman E. Schäffer

**Affiliations:** 1 Institute of Applied Physics, University of Tübingen, Tübingen, Germany; 2 Department of Physiology, University of Tübingen, Tübingen, Germany; 3 Department of Cardiology and Cardiovascular Diseases, University of Tübingen, Tübingen, Germany

## Abstract

Platelets are small anucleate blood cells involved in haemostasis. Platelet activation, caused by agonists such as thrombin or by contact with the extracellular matrix, leads to platelet adhesion, aggregation, and coagulation. Activated platelets undergo shape changes, adhere, and spread at the site of injury to form a blood clot. We investigated the morphology and morphological dynamics of human platelets after complete spreading using fast scanning ion conductance microscopy (SICM). In contrast to unstimulated platelets, thrombin-stimulated platelets showed increased morphological activity after spreading and exhibited dynamic morphological changes in the form of wave-like movements of the lamellipodium and dynamic protrusions on the platelet body. The increase in morphological activity was dependent on thrombin concentration. No increase in activity was observed following exposure to other activation agonists or during contact-induced activation. Inhibition of actin polymerization and inhibition of dynein significantly decreased the activity of thrombin-stimulated platelets. Our data suggest that these morphological dynamics after spreading are thrombin-specific and might play a role in coagulation and blood clot formation.

## Introduction

Platelets are small cell fragments circulating down the vascular branch^1^. Upon endothelial injury of the blood vessel wall, they become exposed to the subendothelial extracellular matrix, which leads to platelet activation and adhesion^2–5^. Activated platelets aggregate and form a blood clot to close the site of injury and prevent blood loss^6^. During activation, a major reorganization of the platelet cytoskeleton is induced^7–10^, leading to the transition from a discoid platelet shape to a more spherical shape^11^. The marginal band of microtubules, which maintains the discoid shape of the resting platelet^12, 13^, expands after activation, driven by the microtubule motor dynein^14^. Activated platelets develop filopodia and lamellipodia^15^ promoting adhesion and spreading on a substrate^16–18^. During spreading, the actin network remodels and develops new actin fibers in order to maintain the shape of the spread platelet^9, 19^. Microtubules in the spread platelet reorganize and redistribute in the cytoplasm^20^ to support the secretion of granules stored inside the platelet to the extracellular space^21^. Granula constituents propagate subsequent activation, adhesion, and spreading of platelets and ultimately the formation of a blood clot.

In this study we investigated the influence of different activation agonists on the morphology of spread human platelets. We visualized and quantified morphological dynamics of spread platelets with high spatial and temporal resolution using scanning ion conductance microscopy (SICM)^22^, a non-contact scanning probe microscopy technique excellently suited for imaging the topography of living cells^23–25^. The contact-free imaging mechanism of SICM allowed us to investigate platelets without mechanical interference, thus avoiding an additional mechanical activation stimulus^26, 27^. SICM has been used for imaging living platelets^28, 29^, for investigating the shape^30^ and the spreading process^31^ of platelets, and for measuring their mechanical properties during activation^32^.

We found that thrombin-stimulated platelets exhibit highly dynamic changes in their morphology after completing the spreading process. These dynamics were dependent on thrombin concentration and did not occur in platelets stimulated with other agonists or in platelets activated by contact with various substrates. The dynamics in thrombin-stimulated platelets ceased following inhibition of actin polymerization and following inhibition of dynein, while the integrity of microtubules seemed to play a minor role. Our data suggest that these dynamics in spread platelets are thrombin-specific and might affect coagulation when high thrombin concentrations occur during blood clot formation.

## Results

### Thrombin stimulation induces morphological dynamics in platelets after spreading

**Figure 1:**
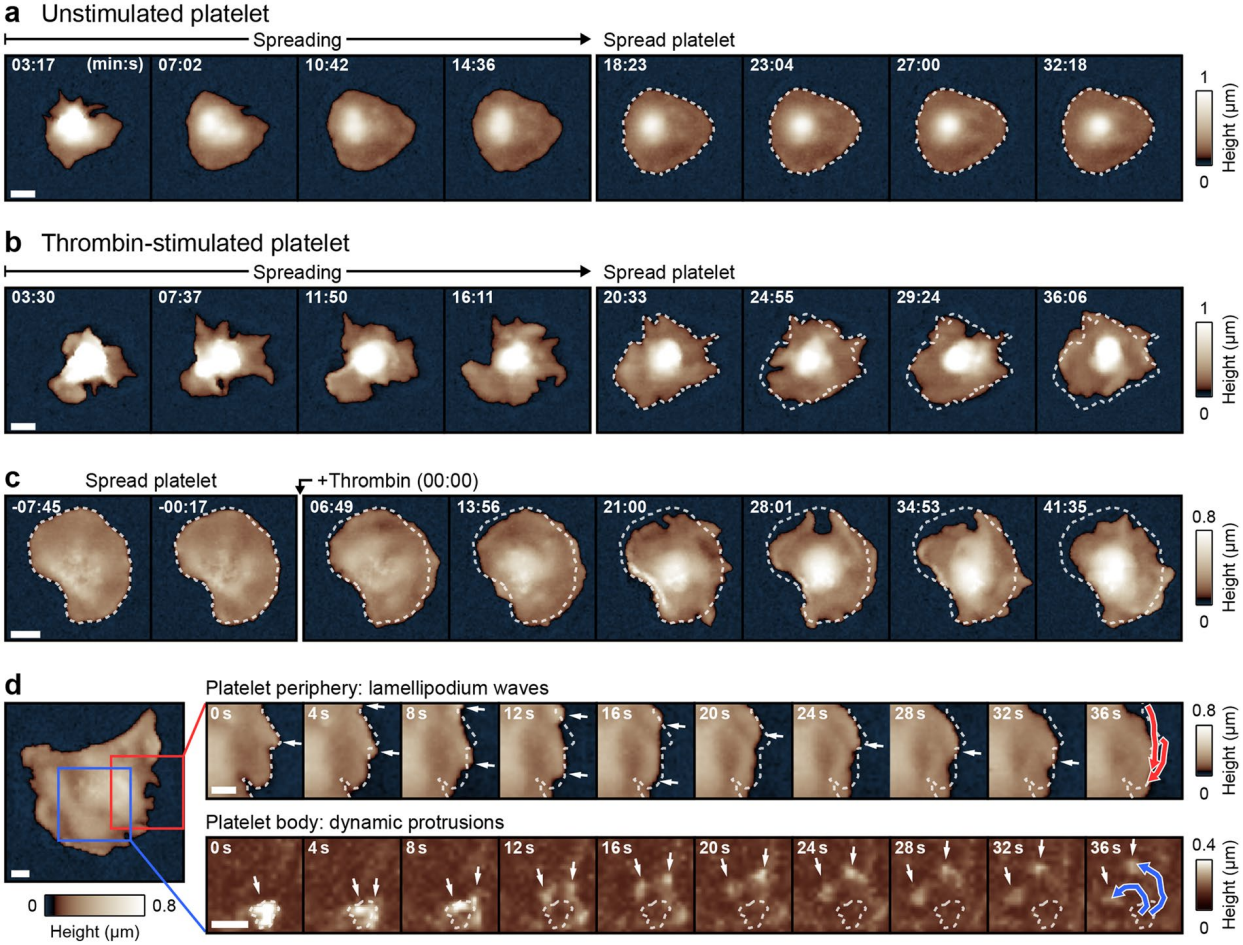
Dynamic morphology of thrombin-stimulated platelets. (**a**) SICM topography image sequence of the spreading process of an unstimulated human platelet (contact with the surface at *t* = 00:00). After the spreading process (at about 20 minutes), the morphology did not change remarkably. (**b**) Image sequence of the spreading process of a human platelet stimulated with 0.5 U/mL thrombin prior to contact with the surface. The platelet morphology showed dynamic changes after the spreading process was completed. (**c**) Image sequence of an unstimulated spread platelet with the addition of 0.5 U/mL thrombin at *t* = 00:00. The platelet morphology became dynamic after the addition of thrombin. Before the addition of thrombin, the platelet has been allowed to adhere and spread for 30 minutes to complete the spreading process. (**d**) Overview image and high-speed image sequences of the platelet periphery (top row) and of the platelet body (bottom row) of a spread thrombin-stimulated platelet. The high-speed image sequences were recorded 1 and 9 min, respectively, after the overview image. Two modes of dynamics were observed: lamellipodium waves at the platelet periphery (top row, arrows, red traces in the last image show the path of motion) and dynamic protrusions on the platelet body (bottom row, arrows, blue traces in the last image show the path of motion). The dashed lines show the initial platelet edge (top row) or the initial protrusions (bottom row). To increase the contrast of the protrusions, the large-scale curvature of the platelet body was removed (bottom row, s. “Methods”). The full image sequences are provided as Supplementary Movies S1–3. Scale bars: 2 µm (**a**–**c**), 1 µm (**d**).

We imaged the spreading process of platelets without and with thrombin stimulation prior to contact with the surface. Unstimulated platelets spread until they reached a roundish shape (Fig. 1a, Supplementary Movie S1a). The spreading process was typically completed within 20 min (Supplementary Fig. 1a), after which the platelet morphology had reached a steady state and no further remarkable changes were observed. Platelets stimulated with thrombin prior to contact with the surface showed a continuously changing dynamic morphology after spreading (Fig. 1b, Supplementary Movie S1b), although the spreading area did not further increase (Supplementary Fig. 1b). The morphology of unstimulated spread platelets also became dynamic after additional stimulation with thrombin (Fig. 1c, Supplementary Movie S2), indicating that the thrombin-induced dynamics were independent of the spreading process. High-speed SICM imaging revealed two distinct modes of dynamics: (1) Wave-like movements of the lamellipodium in the platelet periphery (Fig. 1d, top row, white arrows, Supplementary Movie S3a) and (2) motion of small protrusions on the platelet body (Fig. 1d, bottom row, white arrows, Supplementary Movie S3b). The lamellipodium waves moved along the platelet edge (Fig. 1d, red traces in the last image show the path of motion) and sporadically changed their direction (Supplementary Fig. 1c). Occasionally, small protrusions evolved from a disappearing lamellipodium wave (Supplementary Fig. 1d).

### Different modes of dynamics show different velocities

**Figure 2:**
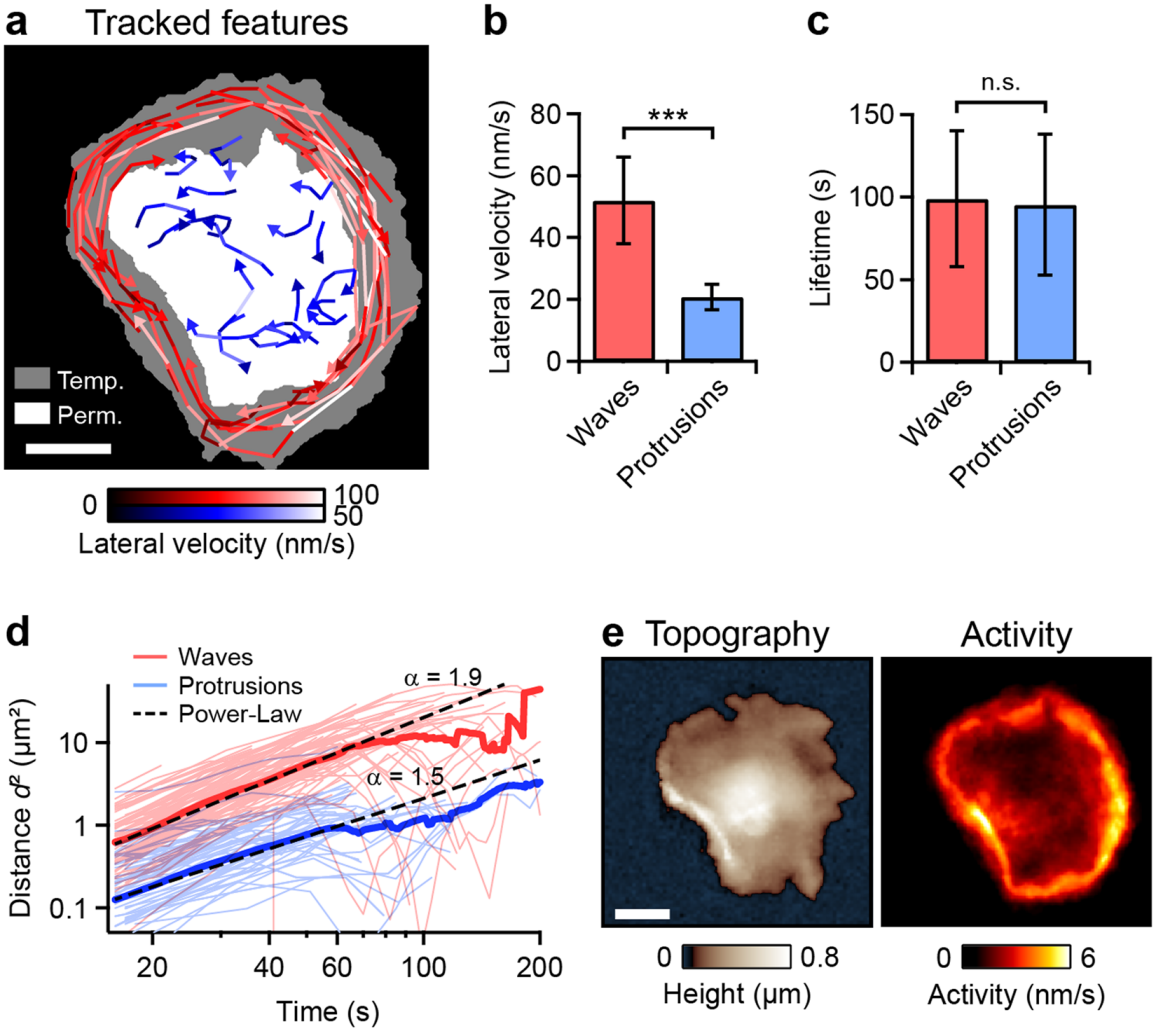
Two modes of dynamics. (**a**) Traces of tracked lamellipodium waves (red) and protrusions (blue) for the thrombin-stimulated platelet shown in Fig. 1c. The brightness of a trace indicates the lateral velocity (s. respective color scale). The areas with the white, grey, and black backgrounds were permanently, temporarily, and never, respectively, covered by the dynamic platelet. (**b**) Average lateral velocity and (**c**) average lifetime of lamellipodium waves (*n* = 72 on 3 different platelets) and protrusions (*n* = 69). (**d**) Mean squared distance (MSD, bold curves) and squared distances of single traces (thin curves) as a function of time, for all tracked lamellipodium waves and protrusions. Each MSD-curve showed a power-law behavior (dashed lines) for times below *t* = 60 s. (**e**) Topography and morphological activity of the platelet shown in panel a. The morphological activity is defined as height change per time (s. “Methods” for details) and shows local changes of the platelet morphology. Areas of high activity correspond to areas with fast and frequently moving features. ***(*P* < 0.001) indicates statistically significant difference. Error bars: standard deviation. Scale bars: 2 µm.

For quantification of their lateral velocity, lamellipodium waves, and protrusions were tracked (Supplementary Fig. 2) in image sequences of thrombin-stimulated platelets for at least 30 min (Fig. 2a). Lamellipodium waves (red traces) were typically located in the platelet periphery. In this region, the dynamic lamellipodium only temporarily covered the substrate (grey colored region). The protrusions (blue traces) were located on the platelet body, which permanently covered the substrate (white colored area). Neither for the lamellipodium waves nor for the protrusions a preferred direction of motion could be identified (s. arrow heads). The lamellipodium waves reached lateral velocities of 30 nm/s to 100 nm/s, whereas the protrusions moved slower with lateral velocities of 10 nm/s to 50 nm/s (s. respective color scale). The average lateral velocity of the lamellipodium waves (50 nm/s) was significantly larger than the average lateral velocity of the protrusions (20 nm/s) (Fig. 2b). The lifetimes of individual waves and protrusions, however, were similar on average (Fig. 2c). The mean squared distance (MSD) traveled by the moving features (lamellipodium waves or protrusions) as a function of time (Fig. 2d, bold curves) was fit to a power-law for times below *t* = 60 s,

$$\langle {d}^{2}\rangle =c\cdot {t}^{\alpha },$$

with distance *d*, pre-factor *c*, time *t*, and power-law exponent *α* (Fig. 2d, dashed lines). The power-law exponent was *α* = 1.9 ± 0.1 for the lamellipodium waves and *α* = 1.5 ± 0.1 for the protrusions. The exponent *α*, which indicates the type of motion (*α* = 2 for directed motion, *α* = 1 for a random walk)^33^, indicates a more directed type of motion for the lamellipodium waves and a more random walk-like type of motion for the protrusions. The breakdown observed for the MSD vs. time relationship for times above 60 s was probably caused by the restricted travelling space owing to the confinement by the platelet edge. Some features changed their direction at higher lifetimes (s. above).

We further used the image sequence to create a map of the morphological activity of the thrombin-stimulated platelet during 30 min after the addition of thrombin (Fig. 2e). The morphological activity is defined as height change per time (s. “Methods” for details) and shows spatially resolved changes of the platelet morphology. Regions with fast and frequently moving features reach higher activity values than regions with slowly moving features. High activity was therefore observed in the platelet periphery, caused by the lamellipodium waves. Lower activity was observed on the platelet body (Supplementary Fig. 3a). We emphasize that the quantitative measure “activity” in this manuscript does not quantify the degree of platelet activation, but rather the amount of morphological dynamics.

### Morphological activity depends on thrombin concentration and is not increased for other activation agonists

**Figure 3:**
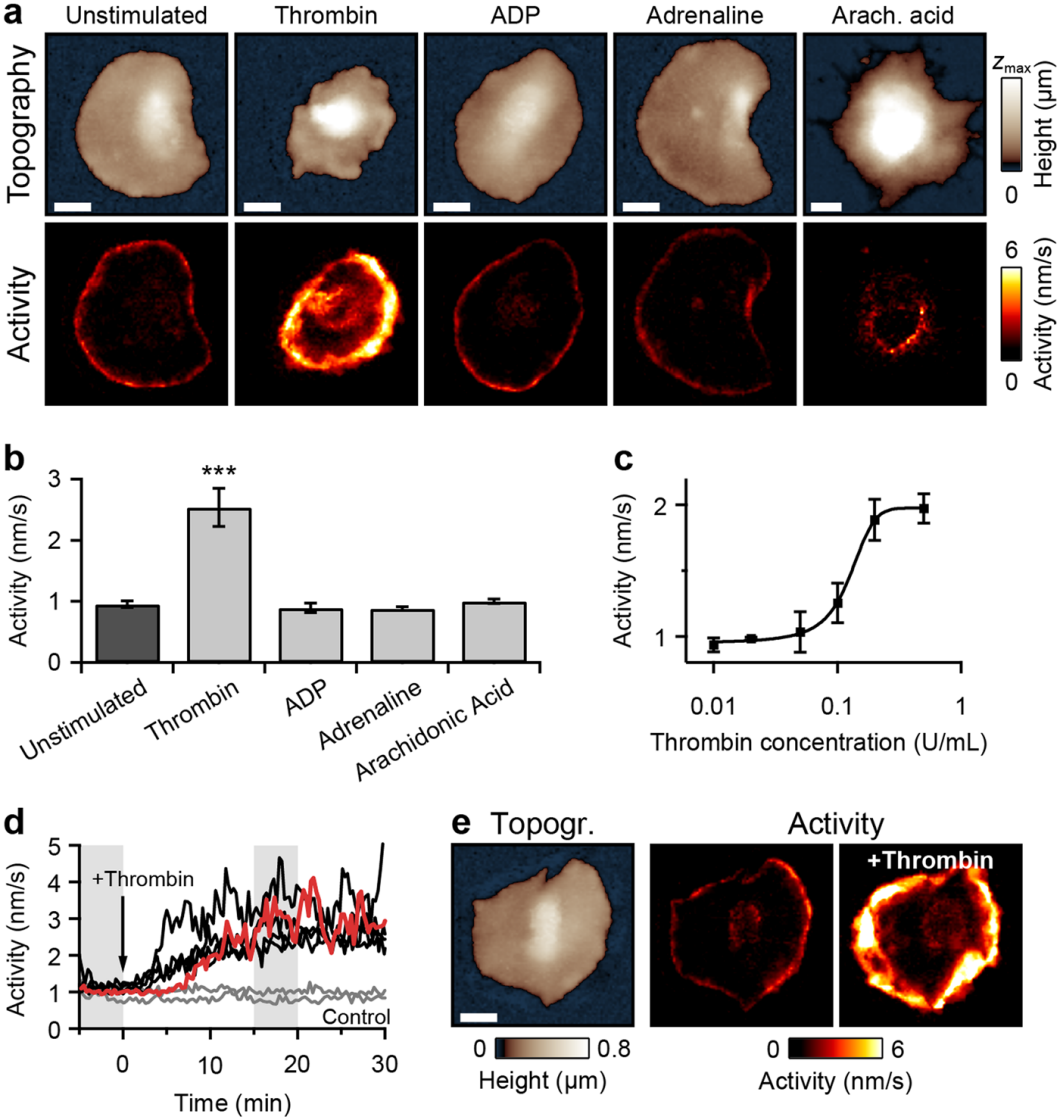
Morphological activity of spread platelets activated with different agonists. (**a**) Topography and activity maps of spread platelets stimulated with different agonists prior to surface contact and spreading. Thrombin-stimulated (0.5 U/mL) platelets showed high morphological activity. Platelets activated only by contact with the surface (unstimulated) or with ADP (20 µM), adrenaline (20 µM) or arachidonic acid (0.5 µM) showed low morphological activity after spreading. Height scale range (*z*_max_): 2 µm for arachidonic acid, 0.8 µm otherwise. (**b**) Average activity of spread platelets activated by surface contact (unstimulated), thrombin, ADP, adrenaline, or arachidonic acid (*n* = 6 platelets each). (**c**) Average activity of thrombin-stimulated spread platelets as a function of thrombin concentration (dose-response relationship) with an EC_50_ of 0.13 U/mL. (**d**) Activity as a function of time for spread platelets with addition of thrombin at *t* = 0 min. The activity substantially increased after this addition, whereas it remained constant after the addition of Tyrode-HEPES buffer (control). Red trace: platelet shown in panel e. (**e**) Topography of the red marked platelet from panel d before the addition of thrombin and activity maps of the platelet during the grey marked time intervals before and after the addition of thrombin. ***(*P* < 0.001) indicates statistically significant difference. Error bars: SEM. Scale bars: 2 µm.

We investigated the influence of additional activation agonists on the morphological activity of spread platelets. Platelets were stimulated with an agonist prior to contact with the surface and were allowed to adhere and spread for 20 min. Platelets stimulated with 0.5 U/mL thrombin showed high activity after spreading (Fig. 3a) and exhibited lamellipodium waves and protrusions as described above. Platelets that were not stimulated or stimulated with 20 µM adenosine diphosphate (ADP) or 20 µM adrenaline before surface contact showed only low activity after spreading, owing to slight movements of the edges (Fig. 3a). These movements stopped upon fixation (Supplementary Fig. 3b). Platelets stimulated with 0.5 µM arachidonic acid (AA) had blurry edges with no activity. Their height was larger compared to the other activation agonists, indicative of a distinct increase in platelet volume. On average, the morphological activity of spread thrombin-stimulated platelets was about two times larger compared to unstimulated or ADP-, adrenaline-, and AA-stimulated platelets (Fig. 3b). The thrombin-induced activity was dependent on thrombin concentration, as shown by a dose-response relationship with an EC_50_ (half maximal effective concentration) of 0.13 U/mL (Fig. 3c). When thrombin was added to spread unstimulated platelets, the morphological activity typically increased within 15 min and then stayed at a high level (Fig. 3d,e). No increase in activity was detected when Tyrode-HEPES buffer was added instead of thrombin (Fig. 3d, control). The average activity of spread platelets stimulated with thrombin prior to spreading was similar compared to platelets stimulated after spreading (*P* = 0.73, *n* = 7, not shown). An influence of the substrate or its coating on the activity of contact-activated platelets was not observed: platelets spread to glass substrate or poly-L-lysine-, collagen-, or fibrinogen-coated polystyrene substrates had low activity after spreading (Supplementary Fig. 4a). For each substrate, the morphological activity of platelets increased after the additional stimulation with thrombin (Supplementary Fig. 4b).

### Inhibition of actin polymerization and inhibition of dynein decrease the morphological activity

**Figure 4:**
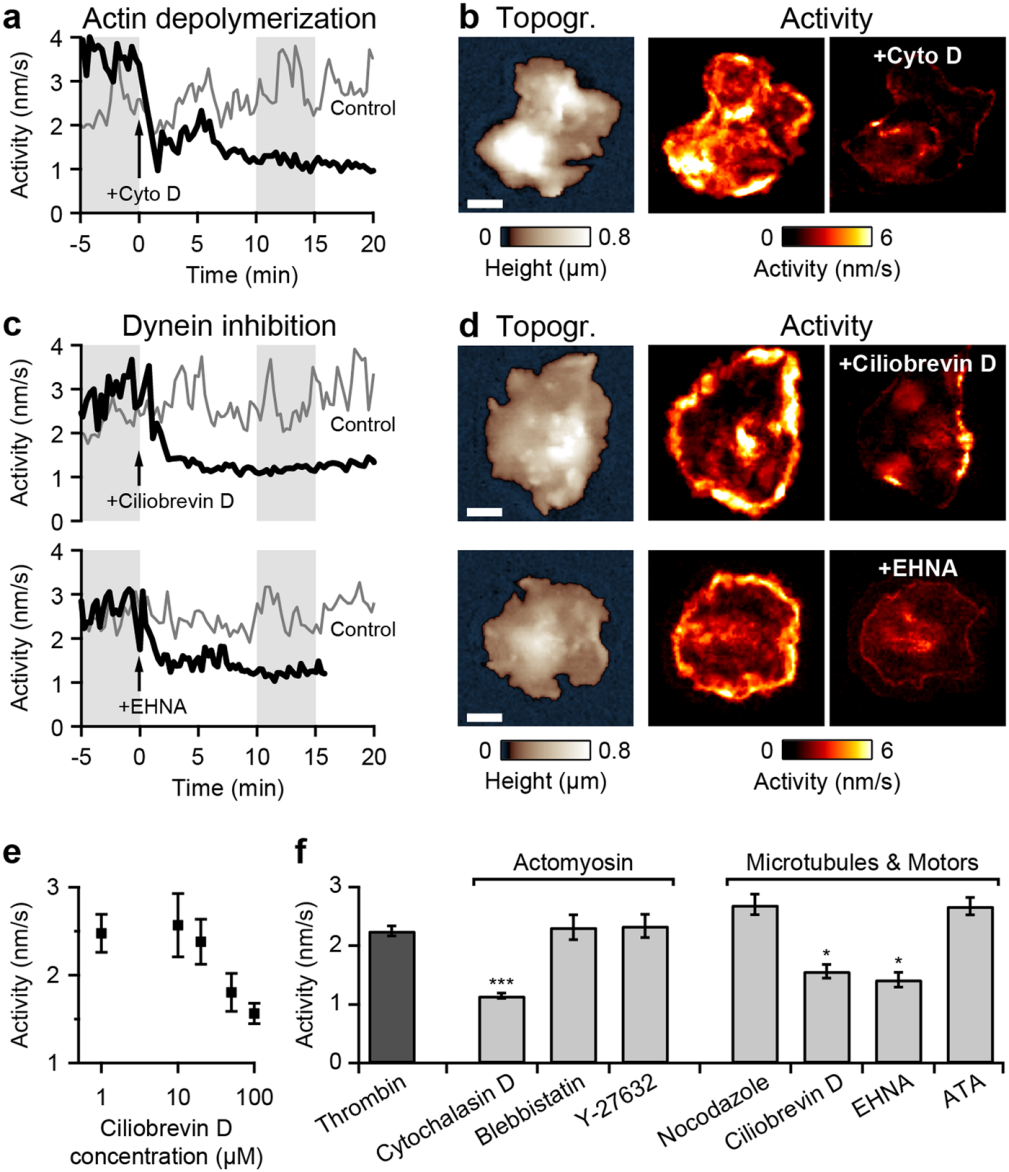
Inhibition of cytoskeleton components and motor proteins in thrombin-stimulated spread platelets. (**a**) Activity as a function of time for a thrombin-stimulated platelet with addition of the actin polymerization inhibitor cytochalasin D (10 µM) at *t* = 0 min. Control: addition of solvent without cytochalasin D. (**b**) Topography and activity maps of the platelet from panel a during the grey marked time intervals before and after the addition of cytochalasin D. (**c**) Activity as a function of time for a thrombin-stimulated platelet with addition of the specific dynein inhibitor ciliobrevin D (100 µM) or the broad-spectrum dynein inhibitor EHNA (1 mM) at *t* = 0 min. Control: addition of solvent without the respective inhibitor. (**d**) Topography and activity maps of the platelets from panel c during the grey marked time intervals before and after the addition of ciliobrevin D or EHNA. (**e**) Average morphological activity as a function of ciliobrevin D concentration. (**f**) Average morphological activity of thrombin-stimulated platelets treated with different cytoskeleton and motor protein inhibitors (*n* = 7 platelets each). In contrast to cytochalasin D, ciliobrevin D, or EHNA, the myosin II inhibitor blebbistatin (100 µM), the ROCK inhibitor Y-27632 (50 µM), the microtubule polymerization inhibitor nocodazole (33 µM), or the kinesin ATPase inhibitor ATA (10 µM) had no significant effect on the activity. The respective image sequences are provided as Supplementary Movies S4 and S5. *(*P* < 0.05) and ***(*P* < 0.001) indicate statistically significant difference. Error bars: SEM. Scale bars: 2 µm.

To identify the cytoskeleton components involved in the dynamics we treated spread thrombin-stimulated platelets with inhibitors of cytoskeleton components and motor proteins. Treatment of platelets with the actin polymerization inhibitor cytochalasin D substantially decreased the morphological activity (Fig. 4a). In the activity maps generated for the grey marked time intervals in Fig. 4a, the decreased activity can clearly be seen (Fig. 4b). Both lamellipodium waves and protrusions disappeared after treatment with cytochalasin D (Supplementary Movie S4a). Further, treatment of thrombin-stimulated platelets with the specific dynein inhibitor ciliobrevin D^34^ or the broad-spectrum dynein inhibitor EHNA^35, 36^ (erythro-9-(2-hydroxy-3-nonyl)-adenine) resulted in a similar decrease of the activity (Fig. 4c and d, Supplementary Movie S4b,c) and in the disappearance of lamellipodium waves and protrusions. The inhibitory effect of ciliobrevin D was effective at about 50 µM and above (Fig. 4e), a dose which effectively inhibits dynein *in vitro* ^34^. Occasionally, treatment with 100 µM ciliobrevin D resulted in rapid formation of filopodia (Supplementary Fig. 5a, arrows), possibly a sign for induced platelet apoptosis^37^. An effect of EHNA at a low concentration of 10 µM was not observed, indicating that phosphodiesterase-2 (PDE2) and adenosine deaminase, which are inhibited by EHNA at this concentration^38–40^, did not affect platelet activity (Supplementary Fig. 5b).

We tested further cytoskeleton inhibitors that affect the actomyosin complex, microtubules, and associated motor proteins. A summary of the effects of all tested inhibitors is given in Fig. 4f. Regarding the actomyosin complex, the morphological activity of thrombin-stimulated platelets was significantly reduced after treatment with cytochalasin D, and was not affected by treatment with the myosin II inhibitor blebbistatin (Supplementary Movie S5a) or the ROCK inhibitor Y-27632 (Supplementary Movie S5b). Regarding microtubules and associated motor proteins, the morphological activity was significantly reduced after treatment with the dynein inhibitors ciliobrevin D or EHNA, and was not significantly affected by treatment with the microtubule polymerization inhibitor nocodazole (Supplementary Movie S5c) or the kinesin ATPase inhibitor ATA^41^ (aurintricarboxylic acid, Supplementary Movie S5d).

**Figure 5:**
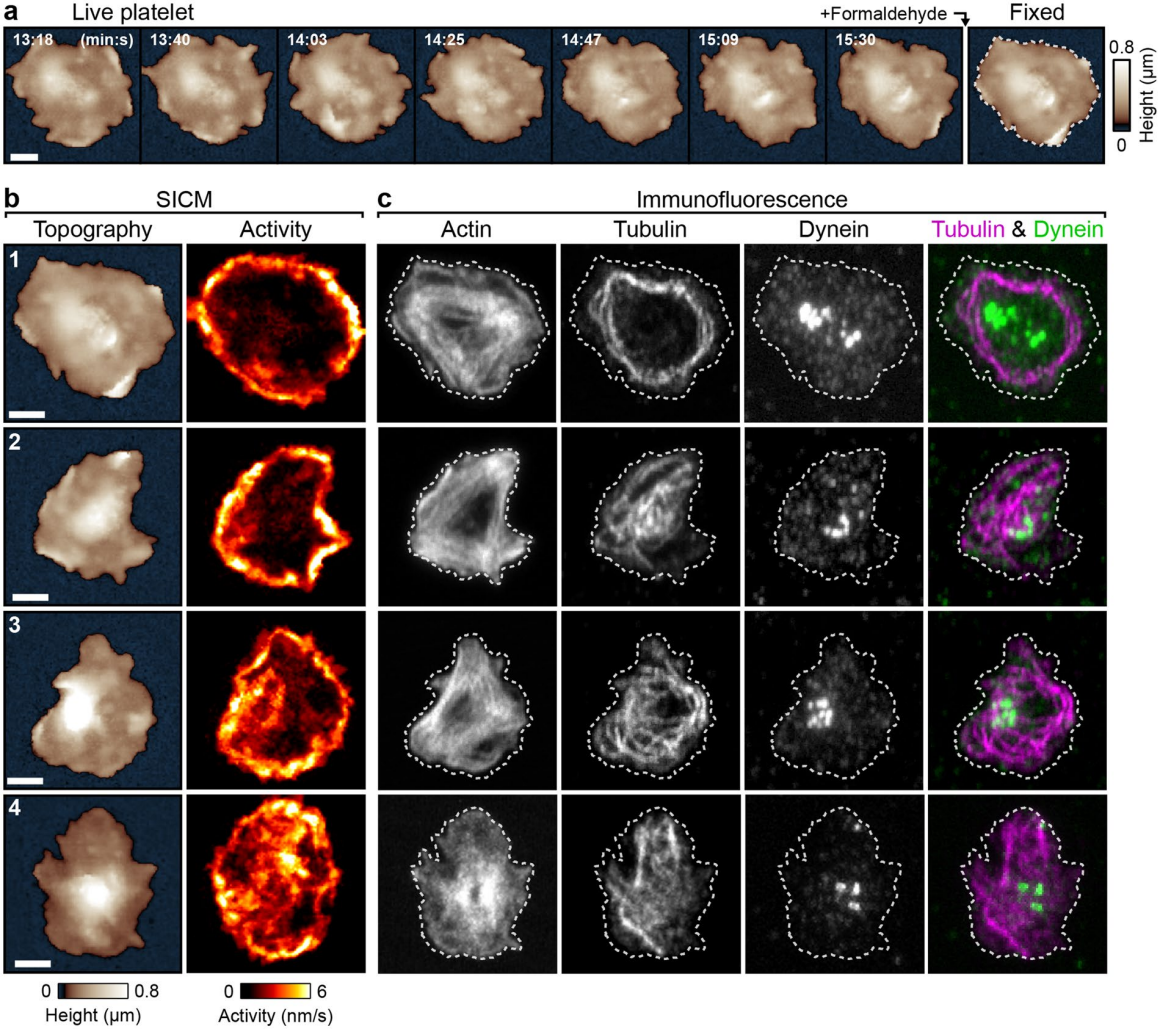
Localization of cytoskeletal components in thrombin-stimulated spread platelets. (**a**) SICM topography image sequence of a thrombin-stimulated platelet before and after fixation with formaldehyde. (**b**) Topography and activity maps of platelets before fixation. (**c**) Confocal fluorescence images of the cytoskeleton components F-actin, α-tubulin and dynein intermediate chain in the fixed platelets. The dashed white lines indicate the outlines of the fixed platelets in the respective SICM topography images. Scale bars: 2 µm.

To determine the intracellular arrangement of the cytoskeletal components involved in the dynamics, we fixed spread thrombin-stimulated platelets during SICM imaging (Fig. 5a, Supplementary Fig. 6, Supplementary Movie S6) to enable subsequent fluorescent labeling of multiple cytoskeleton components. The activity was calculated from the image sequences before fixation (Fig. 5b). After fixation, the platelets were stained for F-actin, α-tubulin, and dynein intermediate chain and confocal fluorescence images were recorded. Actin was present in the platelet body as well as in the lamellipodium (Fig. 5c). Tubulin was visible with clear structure, indicating the presence of intact microtubules (platelets 1–3), or was distributed in the cytoplasm without clear structure (platelet 4). The degree of microtubule fragmentation increased with time after thrombin stimulation (Supplementary Fig. 7). In all cases, dynein was distributed in a spot-like pattern, mainly located at the platelet centers, and was not co-localized with tubulin.

## Discussion

We used SICM to image live platelets during and after spreading with high spatial and temporal resolution. Unlike unstimulated platelets, thrombin-stimulated platelets showed a dynamically changing morphology after spreading (Fig. 1, Supplementary Fig. 1). These thrombin-induced dynamics were independent of the spreading process, as they were also triggered by addition of thrombin to already spread platelets. This non-physiological configuration, however, allowed investigating the thrombin-induced dynamics separately from the morphological changes that occur during spreading.

We observed two distinct modes of dynamics in thrombin-stimulated platelets: wave-like movements of the lamellipodium and motion of protrusions on the platelet body. Wave-like movements of the lamellipodium during platelet spreading have been observed before and were associated with an increased intracellular calcium concentration^42^, which is an indicator for platelet activation^43^. Wave-like movements of the lamellipodium in spread nucleated cells were associated with myosin^44^ or actin polymerization^45–47^. Myosin inhibition by blebbistatin or ROCK inhibition by Y-27632 did not affect the thrombin-induced dynamics in spread platelets, although myosin inhibition during spreading has been shown to alter the shape of spread platelets^48, 49^. This discrepancy might indicate that the thrombin-induced dynamics rely on a different cytoskeletal mechanism than platelet spreading.

In contrast, actin and dynein contributed to the thrombin-induced dynamics in spread platelets, as shown by treatment with the actin polymerization inhibitor cytochalasin D and the dynein inhibitors ciliobrevin D and EHNA (Fig. 4). A functional crosstalk between the actin cytoskeleton and microtubules, possibly mediated by motor proteins, occurs in non-adherent platelets after thrombin stimulation^50^. Disc-to-sphere transition in activated platelets could be inhibited by treating platelets with either cytochalasin D or EHNA^14^. The similar response of spread platelets to cytoskeleton inhibitors in our experiments suggests that the thrombin-induced dynamics in spread platelets and the disc-to-sphere transition are based on a similar cytoskeletal mechanism. The integrity of microtubules, however, may not be essential for the dynamics in spread platelets, as the dynamics also occurred late after thrombin stimulation (>30 min), when microtubules were in a more fragmented state, redistributed in the cytoplasm (Supplementary Fig. 7). Nocodazole-induced disruption of microtubules did not decrease but slightly increased the morphological activity. The microtubule motor dynein was accumulated at the platelet center and the majority of dynein was not associated with microtubules or with the actin cytoskeleton. Possibly, a large fraction of dynein in spread platelets is bound to vesicles or granula located at the platelet center^51^. The effects of dynein inhibition on the morphological activity indicate a functional role of dynein for the dynamics in spread platelets, but the underlying mechanisms of these effects remain unclear. Interactions between dynein and the actin cytoskeleton are known from nucleated cells: In neuronal cells, dynein-generated forces drive axonal growth by influencing the actin network^52–55^. In megakaryocytes, proplatelet formation is driven by dynein, sliding microtubules against the actin network^36, 56^.

Lamellipodium waves moved with a higher lateral velocity (50 nm/s) and in a more directed type of motion than the protrusions (20 nm/s) (Fig. 2). Similar velocities have been reported for the dynamic movement of fibrinogen receptors on the membrane of spread platelets^57^. Velocities in this range are typical for loaded dynein^58^, while the velocity of unloaded dynein can be up to 10 times higher^59^. Actin polymerization waves typically move with substantially higher propagation velocities (75–200 nm/s)^45–47^. Despite the different lateral velocities, lamellipodium waves and protrusions were similarly affected by cytoskeleton inhibitors and had similar lifetimes. This suggests that waves and protrusions have the same underlying cytoskeletal mechanism. Protrusions on the platelet body might move more freely, as they are not confined by the platelet edge, thereby possibly explaining the different velocities and the more random walk-like motion.

The dynamics occurred when platelets were stimulated with high thrombin concentrations (Fig. 3c, EC_50_ = 0.13 U/mL), which typically induce complete platelet activation^60^ and aggregation^61^. In contrast, stimulation with ADP, adrenaline, or AA or contact of platelets with polystyrene, glass, collagen, or fibrinogen did not induce dynamics. Our data suggest that the dynamics after spreading are thrombin-specific and occur at high thrombin concentrations prevailing inside blood clots during coagulation^62^. In this stage, platelets generate contractile forces^63^, leading to stiffening of the blood clot^64^. The increased morphological activity, which occurs on a similar timescale as the contraction^65^ and softening^32^ of single platelets (about 20 minutes), might facilitate or speed up coagulation and the closure of the injured vessel wall.

## Methods

### Platelet isolation and stimulation

All procedures were approved by the institutional ethics committee (270/2011BO1) and comply with the declaration of Helsinki. Informed consent was obtained from all participants. Human platelets were isolated from freshly drawn blood of healthy volunteers mixed with acid citrate dextrose to prevent coagulation as described^30^. First, platelet-rich plasma (PRP) was gained from whole blood by centrifugation at 250 g for 20 min. Tyrode-HEPES buffer (136.89 mM NaCl, 2.68 mM KCl, 1.05 mM MgCl_2_, 0.42 mM NaH_2_PO_4_, 11.9 mM NaHCO_3_, 1 g/L D-glucose, 1 g/L bovine serum albumin (BSA), 4 mM HEPES), pH 6.5, was added to the PRP at a ratio of 1:1. Remaining red and white blood cells were removed with a second centrifugation step (100 g for 20 min). The platelet containing supernatant was centrifuged at 900 g for 10 min. The resulting supernatant was then discarded and the platelet pellet was re-suspended in Tyrode-HEPES buffer, pH 7.4. For SICM measurements, about 10^8^ platelets were added to a “TC”-treated (“tissue culture”) polystyrene culture dish (Greiner Bio-One, Frickenhausen, Germany) containing Tyrode-HEPES buffer and allowed to adhere and spread. For the experiments in Figs 1a,b and 3a,b, and Supplementary Fig. 7a,b, platelets were activated in solution before adhesion and spreading with thrombin (Roche Applied Science, Penzberg, Germany), adenosine diphosphate (ADP, Sigma Aldrich, St. Louis, Missouri, USA), arachidonic acid (AA, Sigma Aldrich) or adrenaline (Sigma Aldrich). For all other experiments, platelets were allowed to spread before stimulation with thrombin, which allowed the investigation of the thrombin-induced dynamics separately from the spreading process. Unless stated otherwise, 0.5 U/mL thrombin was used for stimulation. For platelet spreading experiments, platelet suspension was added directly to the cell culture dish and washed away after 15 seconds. The adherent platelets were then allowed to spread. For some experiments, the culture dishes were coated with collagen (10 µg/cm²; Takeda, Linz, Austria), fibrinogen (0.1 mg/mL; Sigma Aldrich) or poly-L-lysine (0.1 mg/mL; Sigma Aldrich) before adding platelets.

### Inhibition and immunofluorescence of cytoskeletal components

The following cytoskeleton inhibitors were used at the given concentrations, unless stated otherwise: Cytochalasin D (Sigma Aldrich) solved in dimethyl sulfoxide (DMSO) for inhibition of actin polymerization at 10 µM final concentration; blebbistatin (Abcam, Cambridge, UK) solved in DMSO for myosin II inhibition at 100 µM final concentration; Y-27632 (Sigma Aldrich) solved in DMSO for inhibition of rho-associated protein kinase (ROCK) at 50 µM final concentration; nocodazole (Sigma Aldrich) for inhibition of microtubule polymerization at 33 µM final concentration; EHNA^35^ (erythro-9-(2-hydroxy-3-nonyl)-adenine; biomol, Hamburg, Germany) solved in DMSO for dynein inhibition at 1 mM final concentration or ciliobrevin D^34^ (Merck Millipore, Billerica, Massachusetts, USA) solved in DMSO for inhibition of dynein ATPase; ATA^41^ (aurintricarboxylic acid; Sigma Aldrich) solved in ethanol for kinesin inhibition at 10 µM final concentration.

For immunofluorescence measurements, “TC”-treated glass bottom culture dishes were used (Greiner Bio-One). Platelets were fixed in Tyrode-HEPES buffer containing 2% formaldehyde for 5 min during SICM imaging. Afterwards, the platelets were post-fixed for 5 min with ice-cold ethanol at −20 °C. The platelets were rehydrated in phosphate buffered saline (PBS) for 30 min and then blocked with 1% BSA in PBS for 10 min. The following antibodies and concentrations were used for staining cytoskeletal components with 0.1% BSA in PBS for 60 min at room temperature: Anti α-tubulin monoclonal mouse antibody, Alexa Fluor 594 conjugate (diluted 1:200, clone DM1A; Abcam); anti dynein intermediate chain monoclonal mouse antibody, Alexa Fluor 647 conjugate (diluted 1:50, clone 74-1; Santa Cruz Biotechnology, Dallas, Texas, USA); phalloidin iFluor 488 conjugate (diluted 1:1000, CytoPainter; Abcam). Confocal fluorescence images were recorded with a laser scanning confocal microscope (C2; Nikon, Tokyo, Japan) using a 100 × oil immersion objective and Nikon Elements AR software.

### Expression of extracellular P-selectin

To determine the degree of activation, the expression of extracellular P-selectin of unstimulated and thrombin-stimulated spread platelets was quantified by immunofluorescence. Two separate compartments containing either unstimulated or thrombin-stimulated platelets in one dish were created using two-well culture inserts (ibidi, Martinsried, Germany). Platelets were fixed in PBS containing 2% formaldehyde for 10 min after removal of the culture insert with sterile tweezers. Fixed platelets were blocked with 1% BSA in PBS for 10 min and incubated for 60 min with PBS containing anti CD62P/P-selectin monoclonal mouse antibody, PE conjugate (A16339; Molecular Probes, Thermo Fisher, Waltham, Massachusetts, USA), dilution 1:200. Epifluorescence images of platelets in both compartments were recorded with the same exposure time. The images were then analyzed using the CellProfiler software^66, 67^ and the average fluorescence intensity was measured for each individual platelet, indicating its degree of activation.

### SICM imaging and activity mapping

A custom-built SICM setup (Supplementary Fig. 8) was used to image the topography of adherent platelets in backstep/hopping mode^24, 68^ with high temporal and spatial resolution. The setup consisted of a 200 µm *xy*-scanner (P-542.2CL; Physik Instrumente, Karlsruhe, Germany) for lateral positioning of the sample, a 15 µm *z*-scanner (P-753.11C; Physik Instrumente) for fast vertical positioning of the nanopipet and a patch clamp amplifier (EPC-800; HEKA Elektronik, Lambrecht, Germany) for ion current measurement. The setup was mounted on an inverted optical microscope (Ti-U; Nikon) for optical access to the nanopipet and the sample. Borosilicate nanopipets with a typical inner diameter of 80 nm were fabricated using a CO_2_-laser-based micropipet puller (P-2000; Sutter Instrument, Novato, CA, USA). Topography images were recorded with an ion current trigger of 99.5% of the free ion current and a constant pipet approach and retract speed of 340 µm/s. A pixel resolution of 125 nm/pixel was chosen for all images to fully utilize the lateral imaging resolution of 1.5 times the inner diameter of the nanopipet^69, 70^. Image sequences were recorded with an average duration of 4 seconds per image consisting of 32 × 32 pixels in a 4 × 4 µm² scan area or with an average duration of 17 seconds per image consisting of 80 × 80 pixels in a 10 × 10 µm² scan area. To increase the contrast of protrusions on the platelet surface, the large-scale curvature of the platelet surface was removed in some topography images (Fig. 1d, bottom row, Supplementary Fig. 2b, Supplementary Movie S3b) by applying a “rolling ball” background removal filter^71^.

For visualization and quantification of fast changes in platelet morphology, we calculated maps of the absolute height change between two consecutive images in an image sequence, divided by the respective image duration, termed as maps of the momentary “morphological activity” of the platelet. Activity maps that are insensitive to temporal variations of the momentary activity were calculated by averaging all momentary activity maps within a time interval of interest (5 min unless stated otherwise). In the obtained activity maps, regions of the platelet with fast and frequent movements during that time interval show higher activity values than regions with slow movements. A global value for the activity of the whole platelet was then gained by averaging all local values within the platelet area. The global values of the momentary activity maps were used in the graphs displaying the time dependence of the activity. The global values of the averaged activity maps were used to compare the activity before and after the addition of substances. For activation and cytoskeleton inhibitor measurements, the activity was determined during 5 min before and 15–20 min (activators) or 10–15 min (inhibitors) after the addition of the respective substance.

### Feature position tracking

For tracking the position of moving features in the image sequences, images of the height difference between two consecutive images were calculated (Supplementary Fig. 2). Appearing or disappearing features were identified as areas with positive or negative values in the height difference images. Moving features were identified as adjacent areas of positive and negative values. The point of the largest positive height change was defined as the momentary position of the tracked feature.

### Statistics

Data are presented as arithmetic means ± SEM (standard error of the mean), unless stated otherwise. All results were tested using Tukey’s test. Results were considered significantly different for *P*-values < 0.05.

## Electronic supplementary material

Supplementary Movie S1

Supplementary Movie S2

Supplementary Movie S3

Supplementary Movie S4

Supplementary Movie S5

Supplementary Movie S6

Supplementrary PDF File
