# Supplementary material for: Thrombin-induced cytoskeleton dynamics in spread human platelets observed with fast scanning ion conductance microscopy
Source: Sci Rep. 2017 Jul 6;7:4810. doi: 10.1038/s41598-017-04999-6 (PMC5500533; doi:10.1038/s41598-017-04999-6)
Supplement: Supplementary file 7 — Supplementrary PDF File [file 41598_2017_4999_MOESM7_ESM.pdf]

Thrombin-induced cytoskeleton dynamics in spread human platelets observed with fast scanning ion conductance microscopy – Supplementary information

Jan Seifert,[1] Johannes Rheinlaender,[1] Florian Lang,[2] Meinrad Gawaz,[3] and Tilman E. Schäffer[1]*

[1] *Institute of Applied Physics, University of Tübingen, Germany*

[2] *Department of Physiology, University of Tübingen, Germany*

[3] *Department of Cardiology and Cardiovascular Diseases, University of Tübingen, Germany*

Supplementary Figures

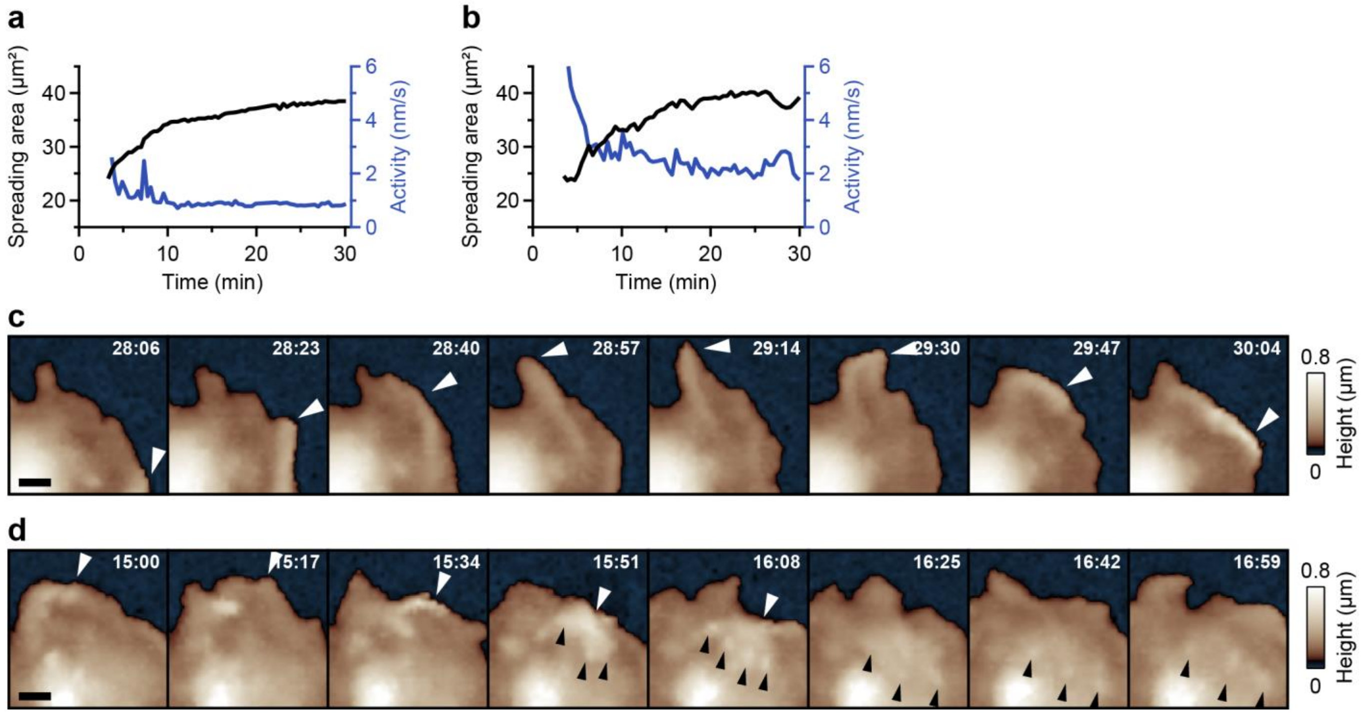

Supplementary Figure 1: Platelet spreading and morphological dynamics. (a) Spreading area and activity of the unstimulated platelet shown in Fig. 1a and (b) of the thrombin-stimulated platelet shown in Fig. 1b as a function of time. The spreading process was typically completed after 20 min (when the increase in spreading area within 5 min was smaller than area fluctuations). (c) Lamellipodium wave (white arrows) changing direction. (d) Dynamic protrusions (black arrows) evolving from a lamellipodium wave (white arrows). Scale bars: 1 µm.

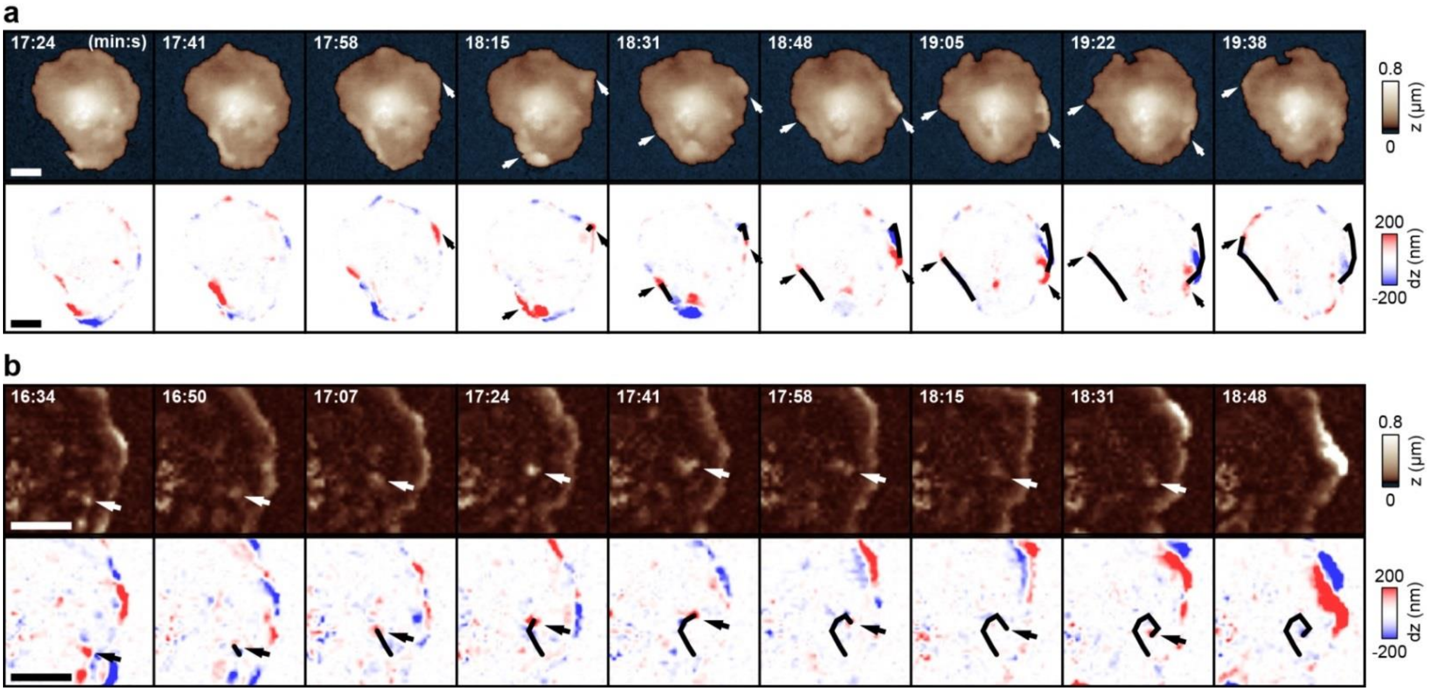

Supplementary Figure 2: Feature position tracking. Topography image sequences (top rows) and frame-wise height difference images (bottom rows), showing (a) lamellipodium waves and (b) dynamic protrusions on the activated platelet from Fig. 2. Moving features were identified in the difference images as positive (red color) and negative (blue color) values. Scale bars: 2 µm.

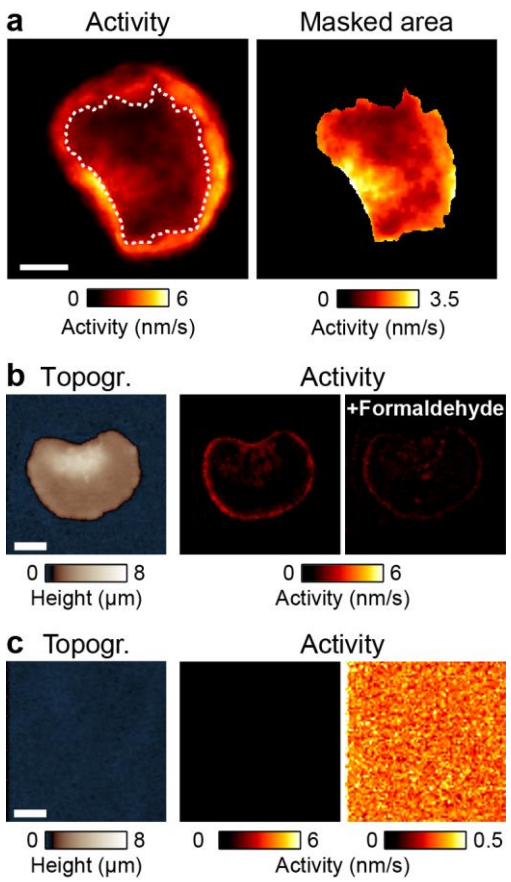

Supplementary Figure 3: Control measurements. (a) Activity map shown in Fig. 2e and activity on the platelet body (masked area) shown with increased contrast. (b) Topography of a resting platelet before fixation and activity maps before and after fixation with 2% formaldehyde. The activity almost completely vanished after fixation. The remaining faint halo after fixation is probably caused by the experimental uncertainty in detecting the edge of the platelet. (c) Topography and activity maps of a polystyrene substrate without a platelet. The average activity on the substrate is 0.3 nm/s ± 0.07 nm/s (SD). Scale bars: 2 µm.

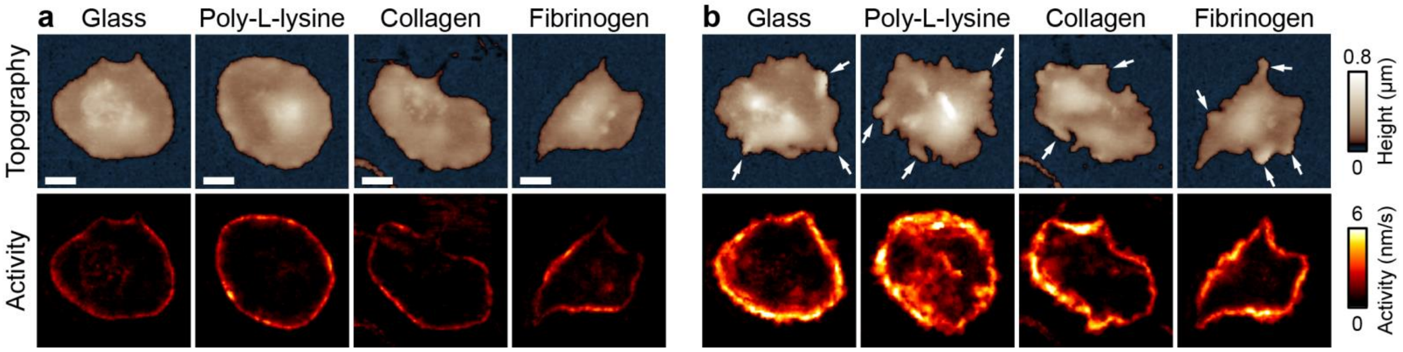

Supplementary Figure 4: Contact activation and thrombin stimulation on different substrates. (a) Topography and activity maps of fully spread platelets on glass, poly-L-lysine, collagen, and fibrinogen substrates. (b) Same platelets as in panel a after additional stimulation with thrombin. Increased morphological activity and lamellipodium waves (white arrows) were observed after the addition of thrombin. Scale bars: 2 µm.

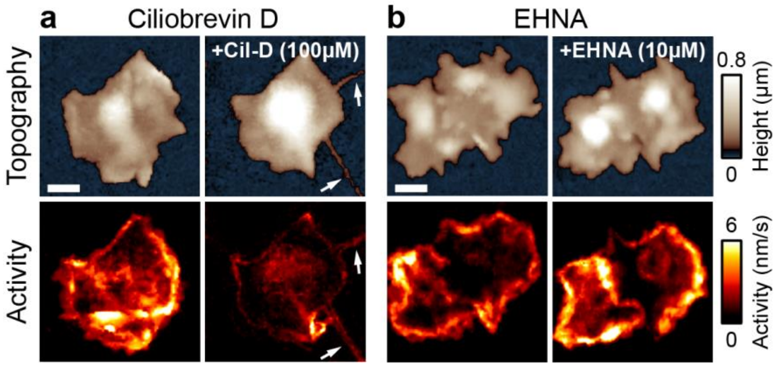

Supplementary Figure 5: Inhibition of dynein. (a) Topography and activity map of a thrombin-stimulated platelet before and after the addition of 100 µM ciliobrevin D. Rapid formation of filopodia (arrows) was observed, possibly a sign of apoptosis. (b) Topography and activity maps of a thrombin-stimulated platelet before and after the addition of 10 µM EHNA. No decrease in morphological activity was observed. Scale bars: 2 µm.

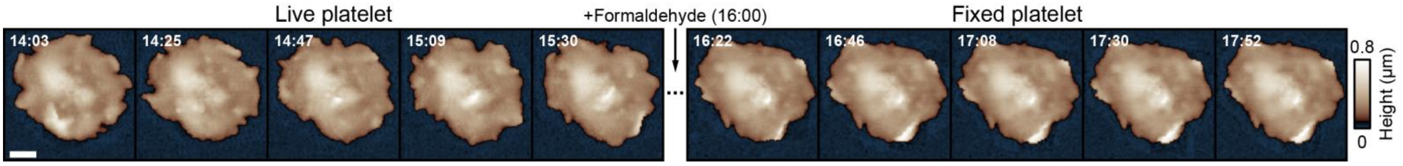

Supplementary Figure 6: Fixation process of the platelet shown in Fig. 5a. The image sequence shows the platelet topography before and after the addition of formaldehyde. The dynamic movements stopped within seconds after the addition of formaldehyde. The full image sequence is provided as Suppl. Movie S6. Scale bar: 2 µm.

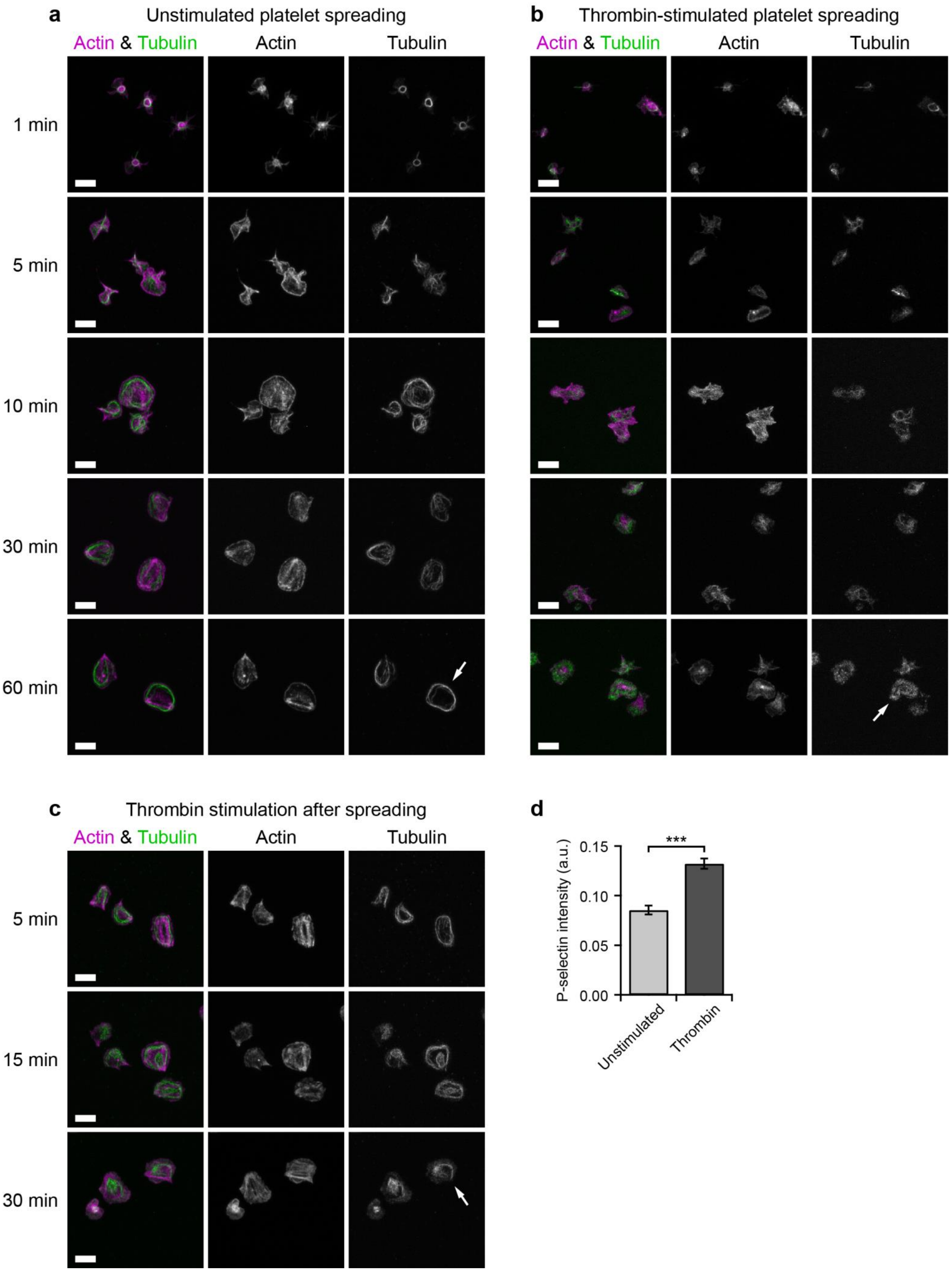
a
Unstimulated platelet spreading
Actin & Tubulin
Actin
Tubulin
1 min
5 min
10 min
30 min
60 min
b
Thrombin-stimulated platelet spreading
Actin & Tubulin
Actin
Tubulin
c
Thrombin stimulation after spreading
Actin & Tubulin
Actin
Tubulin
5 min
15 min
30 min
d
P-selectin intensity (a.u.)
0.15
0.10
0.05
0.00
***
Unstimulated
Thrombin

Supplementary Figure 7: Cytoskeleton components during platelet spreading and expression of extracellular P-selectin. (a) Fluorescence images of actin and tubulin in unstimulated platelets fixed at different times during spreading. Distinct microtubule structures can be observed after completion of the spreading process (arrow). (b) Platelets stimulated with thrombin prior to contact with the surface. Microtubules occur blurrily, indicating a more fragmented state after completion of the spreading process (arrow). (c) Spread platelets with subsequent addition of thrombin. The blurry tubulin signal at $t = 30$ min indicates increased fragmentation of the microtubules (arrow). The platelets have been allowed to adhere and spread for 30 minutes before the addition of thrombin. (d) Expression of extracellular P-selectin for unstimulated (n=47) and thrombin-stimulated spread platelets (n=69) on polystyrene substrate. P-selectin intensity is increased for thrombin-stimulated platelets, indicating a higher degree of activation. Platelets have been allowed to adhere and spread for 30 minutes before fixation. *** ($P < 0.001$) indicates statistically significant difference. Error bars: SEM. Scale bars: 5 µm.

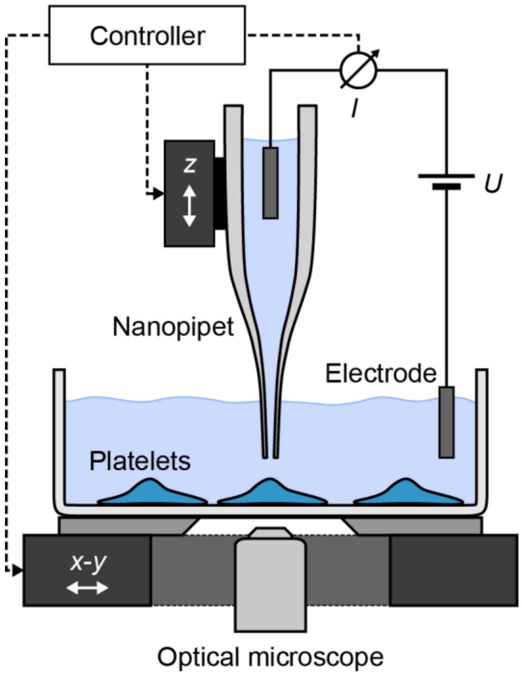

Supplementary Figure 8: Schematic of the SICM setup. An electrolyte-filled nanopipet is scanned across the sample (platelets). The ion current between two electrodes through the tip of the nanopipet depends on the tip-sample distance and is used to determine the height of the sample surface at each lateral position *x*, *y*. An optical microscope allows optical control of the position of the pipet tip in the culture dish.

Supplementary Movies

Movie S1: Spreading process of platelets. Shown is (a) the unstimulated platelet from Fig. 1a and (b) the thrombin-stimulated platelet from Fig. 1b. Scale bar: 2 µm.

Movie S2: Addition of thrombin to a fully spread platelet. Unstimulated platelet shown in Fig. 1c with addition of thrombin at $t = 0$ min. Scale bar: 2 µm.

Movie S3: Lamellipodium waves and protrusions. Zoom-ins on (a) the platelet periphery and (b) the platelet body of the thrombin-stimulated platelet shown in Fig. 1d, top row. Scale bar: 1 µm.

Movie S4: Inhibition of cytoskeleton components. (a) Thrombin-stimulated platelet shown in Fig. 4a and b during inhibition of actin polymerization with 10 µM cytochalasin D (cyto-D). Thrombin-stimulated platelets shown in Fig. 4c and d during inhibition of dynein ATPase with (b) 50 µM ciliobrevin D (Cil-D) and (c) 1 mM EHNA. Scale bar: 2 µm.

Movie S5: Inhibition of cytoskeleton components. Thrombin-stimulated platelets during (a) inhibition of myosin II with 100 µM blebbistatin, (b) inhibition of ROCK with 50 µM Y-27632, (c) inhibition of microtubule polymerization with 33 µM nocodazole, and (d) inhibition of kinesin ATPase with 10 µM ATA. Scale bar: 2 µm.

Movie S6: Fixation of thrombin-stimulated platelet. Platelet shown in Fig. 5a during fixation with 2% formaldehyde (FDH). Scale bar: 2 µm.
